# Sol-Gel Synthesis and Antioxidant Properties of Yttrium Oxide Nanocrystallites Incorporating P-123

**DOI:** 10.3390/ma7096768

**Published:** 2014-09-19

**Authors:** Rebeca Mellado-Vázquez, Margarita García-Hernández, Arturo López-Marure, Perla Yolanda López-Camacho, Ángel de Jesús Morales-Ramírez, Hiram Isaac Beltrán-Conde

**Affiliations:** 1 Departamento de Ciencias Naturales, Universidad Autónoma Metropolitana-Unidad Cuajimalpa, 4871 Avenida Vasco de Quiroga, Colonia Santa Fé Cuajimalpa, Delegación Cuajimalpa de Morelos, C.P. 05300 Mexico, D.F., Mexico; E-Mails: orchidee040811@gmail.com (R.M.-V.); pylopezc@gmail.com (P.Y.L.-C.); hbeltran@correo.cua.uam.mx (H.I.B.-C.); 2 Instituto Politécnico Nacional-Centro de Investigación en Ciencia Aplicada y Tecnología Avanzada (CICATA) Unidad Altamira, Carretera Tampico Puerto Industrial Altamira, Km 14.5 Altamira, C.P. 89600 Tamaulipas, Mexico; E-Mail: arlopezm@ipn.mx; 3 Instituto Politécnico Nacional-Centro de Investigación e Innovación Tecnológica (CIITEC), Cerrada de Cecati s/n, Col. Santa Catarina, Del. Azcapotzalco, C.P. 02250 Mexico, D.F., Mexico; E-Mail: amoralesra@ipn.mx

**Keywords:** yttrium oxide (Y_2_O_3_), nanocrystallites, sol-gel, antioxidant properties

## Abstract

Yttrium oxide (Y_2_O_3_) nanocrystallites were synthesized by mean of a sol-gel method using two different precursors. Raw materials used were yttrium nitrate and yttrium chloride, in methanol. In order to promote oxygen vacancies, P-123 poloxamer was incorporated. Synthesized systems were heat-treated at temperatures from 700 °C to 900 °C. Systems at 900 °C were prepared in the presence and absence of P-123 using different molar ratios (P-123:Y = 1:1 and 2:1). Fourier transform infrared spectroscopy (FTIR) results revealed a characteristic absorption band of Y–O vibrations typical of Y_2_O_3_ matrix. The structural phase was analyzed by X-ray diffraction (XRD), showing the characteristic cubic phase in all systems. The diffraction peak that presented the major intensity corresponded to the sample prepared from yttrium chloride incorporating P-123 in a molar ratio of P-123:Y = 2:1 at 900 °C. Crystallites sizes were determined by Scherrer equation as between 21 nm and 32 nm. Antioxidant properties were estimated by 2,2-diphenyl-1-picrylhydrazyl (DPPH•) assays; the results are discussed.

## 1. Introduction

Nowadays, nanoscience and nanotechnology have a variety of practical applications in which the synthesis of nanostructured materials is a major trend. Since nanotechnology deals with materials or structures at nanometer scales, typically ranging from subnanometers to several hundred nanometers, and it is well known that the properties are not the same at the nanometer scale as those observed for bulk materials, it has been an aim to produce highly specialized new systems [1]. Nanomaterials are present in different areas as electronic, automotive, construction, biomaterials, and many others [2,3]. For biomaterials applications, there are many different classes, such as polymers, metallic alloys, ceramics, *etc.* [4] Commonly, ceramic materials are inorganic, non-metallic materials prepared from compounds including a metal and a non-metal material, and may be crystalline or partially crystalline [5]. The final uses of ceramic nanomaterials are varied, depending on their synthesis process, chemical composition, and final presentation (nanoparticles, films, *etc.*). For example, bioceramics can be used in biology and medicine fields, which are designed to elicit a specific physiological behavior and to be used in living organisms [6]. Thus, metal oxides nanoparticles have shown potential scavenger behavior and in particular, yttrium oxide (Y_2_O_3_) is nowadays considered an important compound due to its high free energy to oxide formation from elemental yttrium among known metal oxides [7]. It is known that CeO_2_ and NiO nanoparticles are relatively nontoxic to neutrophiles and macrophages, therefore, these particles protect cells from death due to oxidative stress, and the protection is due to the direct antioxidant properties of nanoparticles [8,9,10]. Y_2_O_3_ presents the same cubic structure of cerium oxide, so it can be considered a candidate for biological applications; so, it is considered that may act as a free radical scavenger due to the free energy of oxide formation from elemental yttrium is among the highest known [11]. This metal oxide is characterized by only small deviations from stoichiometry under normal conditions of temperature and pressure and by absorption of water and carbon dioxide from the atmosphere [7]. Y_2_O_3_ has been prepared by several methods, such as liquid-phase reaction [12], polyol [13], spray pyrolysis [14], homogeneous precipitation [15], and sol-gel [16]. The last one has emerged as one of the most promising processes due to its unique advantages, such as high chemical homogeneity, capacity to achieve several compositions, and the ability to prepare preferential crystal structures [17]. P-123 is a typical non-ionic surfactant, has been widely used in the formation of mesoporosity [18]. It is known that mesoporous structure with high specific surface area and pore volume promote the formation of channels and they are affective loading and delivery of drug molecules. For this reason and advantagesin this paper, we report the structural and antioxidant properties of Y_2_O_3_ prepared by sol-gel method using two different precursors and including P-123 poloxamer. Crystal structural characterization was carried out by X-ray diffraction (XRD). Fourier transform infrared spectroscopy (FTIR) studies were conducted to study the evolution of organic-inorganic compounds. Antioxidant properties were evaluated using 2,2-diphenyl-1-picrylhydrazyl (DPPH•) assays. There are no studies to date about Y_2_O_3_ using P-123 poloxamer nanoparticles produced by sol-gel method and its antioxidant properties.

## 2. Results and Discussion

Y_2_O_3_ materials were prepared at different synthesis conditions, as described in the experimental section. Summary of prepared systems used in this paper is presented in Table 1.

**Table 1:** Yttrium oxide (Y_2_O_3_) systems prepared by sol-gel method, key words and general description.

Sample	Precursor	Matrix	Poloxamer	The molar ratio of P-123:Y	T (°C)	Crystallite size and error (nm)
Y1	Y(NO_3_)_3_	Y_2_O_3_	-	-	700	25.8 ± 0.4
Y2					800	27.0 ± 0.1
Y3					900	26.1 ± 0.2
Y4	YCl_3_		-	-	700	32.1 ± 0.4
Y5					800	29.5 ± 0.3
Y6					900	28.9 ± 0.08
Y7	Y(NO_3_)_3_	-	P-123	1:1	900	21.0 ± 0.1
Y8				2:1	900	27.8 ± 0.1
Y9	YCl_3_		P-123	1:1	900	29.5 ± 0.3
Y10				2:1	900	28.8 ± 0.07

### 2.1. Chemical and Structural Characterization

Typical infrared absorption bands of Y_2_O_3_ synthesized from yttrium nitrate and chloride precursors were analyzed by means of FTIR studies. Systems prepared from addition of P-123 poloxamer into the synthesis process and heat-treated at different temperatures were also analyzed.

**Figure 1:**
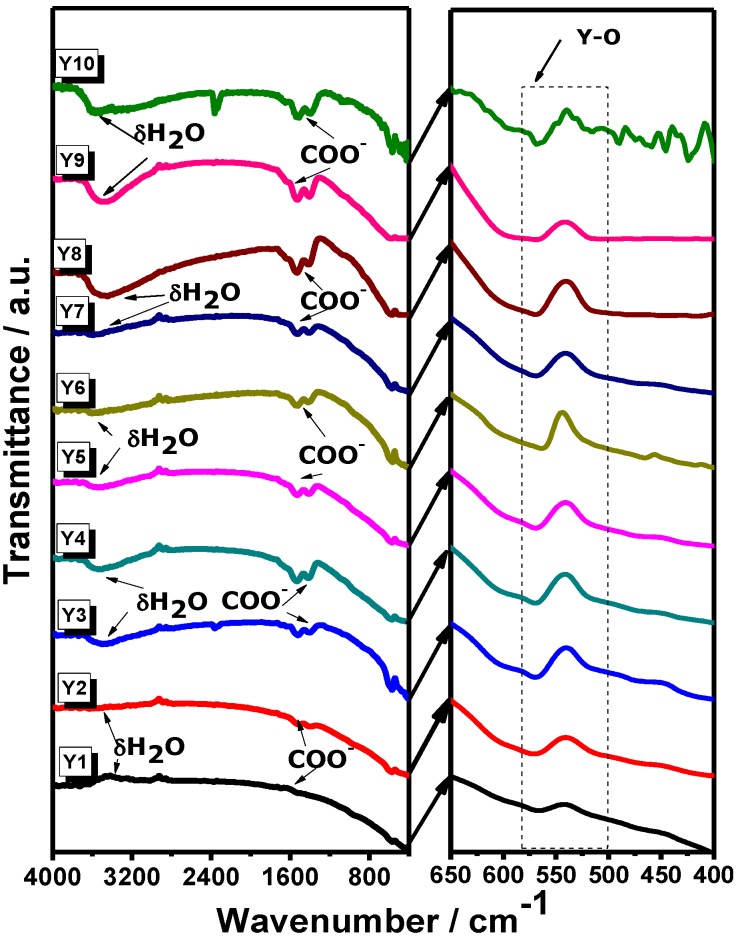
Fourier transform infrared spectroscopy (FTIR) spectra of Y_2_O_3_ systems heat-treated from 700 °C to 900 °C.

**Figure 2:**
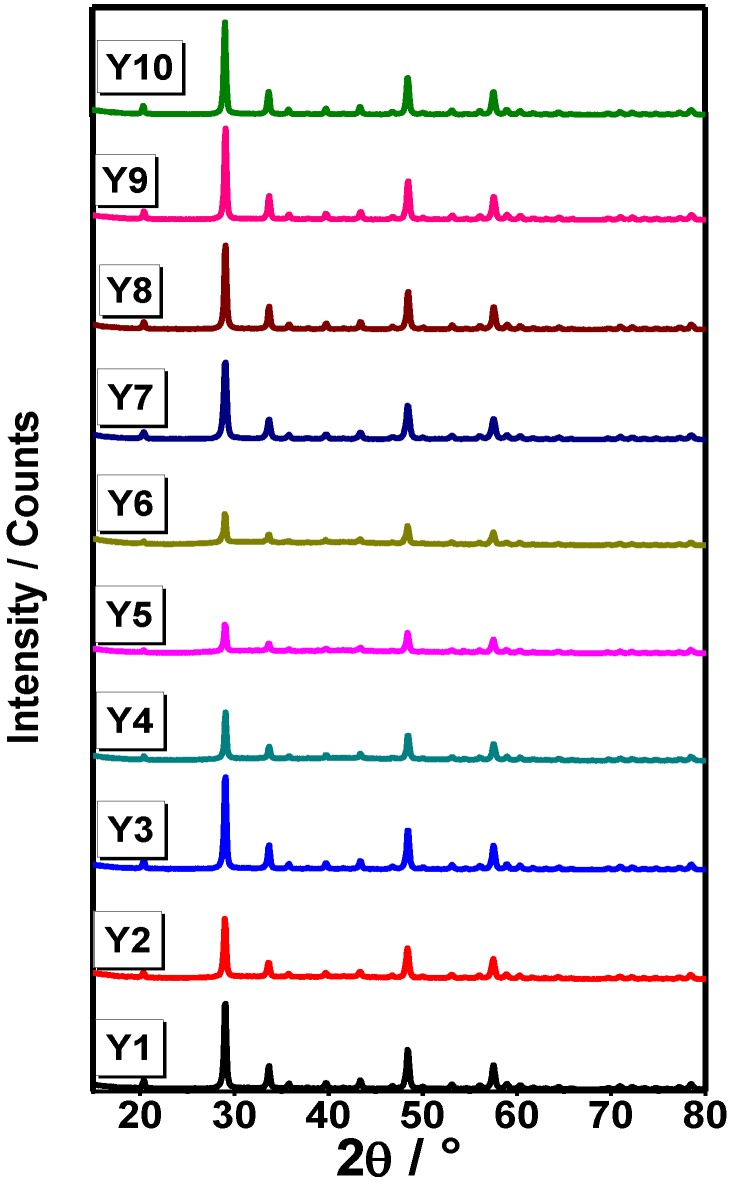
X-ray diffraction (XRD) patterns of Y_2_O_3_ systems heat-treated from 700 °C to 900 °C.

**Figure 3:**
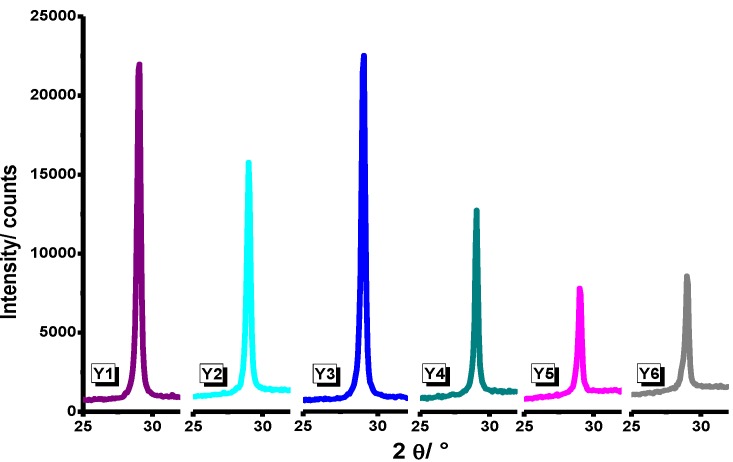
XRD patterns of Y_2_O_3_ systems prepared from yttrium nitrate and yttrium chloride at different temperatures.

In Figure 1, FTIR spectra for Systems Y1–Y10 are presented. For Samples Y1 and Y2, an absorption band is observed around 500 cm^−1^ and 600 cm^−1^, corresponding to oxygen-metal as reported for Y_2_O_3_ when it is prepared by sol-gel method [19]. In Samples Y3–Y10, we observe absorption bands associated to coordinated water (δH_2_O), therefore it can be linked to asymmetric vibration bands corresponding to COO^−^ from air and atmospheric humidity [20]. On the other hand, in Samples Y7–Y10, there is no evidence of (–CH_2_–)_n_– bands at 2875–975 cm^−1^ from the P-123 poloxamer, indicating that it was completely removed after the annealing process at 900 °C. In order to determine the crystalline structure of the samples, an XRD analysis was carried out. XRD profiles of Y_2_O_3_ powders for each synthesis procedure are shown in Figure 2 (Y1–Y12). As can be observed, no secondary phases were found within the detection limit of the XRD technique. It can be seen that the cubic structure of Y_2_O_3_ is formed at 700 °C and it remains stable until 900 °C, Joint Committee on Powder Diffraction Standards Card (JCPDS) 25-1200 [21] with a spatial group Iā3 (lattice parameter 10.604 Å). Samples synthesized from yttrium nitrate (Y1–Y3) showed a greater degree of crystallization compared with those synthesized from yttrium chloride (Y4–Y6); XRD patterns are presented in Figure 3. This effect could be consequence of the thermal decomposition of the metallic precursors during the sol-gel process. As has been already observed for In_2_O_3_ synthesis from nitrate and chloride precursors [22], the first ones are eliminated at lower temperatures, and this behavior would affect the available energy for the crystallization process.

**Figure 4:**
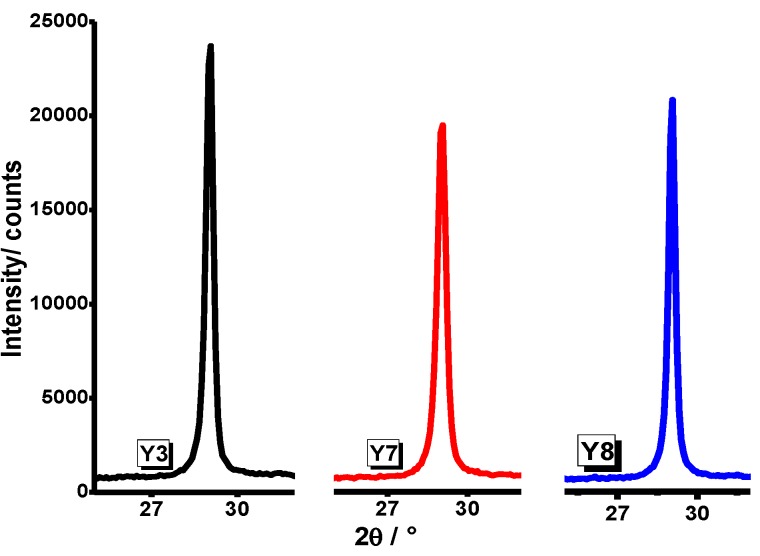
XRD pattern of Y_2_O_3_ powder synthesized from yttrium nitrate in the presence and absence of P-123 poloxamer heat-treated at 900 °C for 1 h.

**Figure 5:**
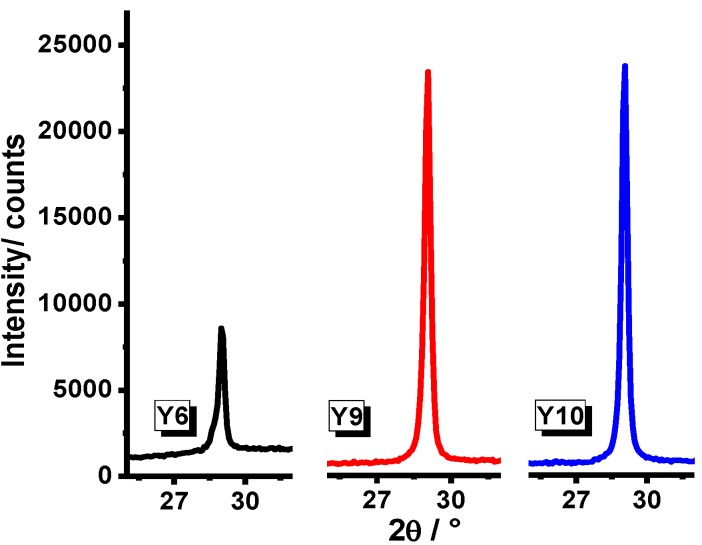
XRD pattern of Y_2_O_3_ powder synthesized from yttrium chloride in the presence and absence of P-123 poloxamer heat-treated at 900 °C for 1 h.

A comparison in crystallization degrees between systems prepared from yttrium nitrate and those prepared from yttrium chloride in the presence and absence of P-123 poloxamer is presented in Figure 4 and Figure 5, respectively.

**Figure 6:**
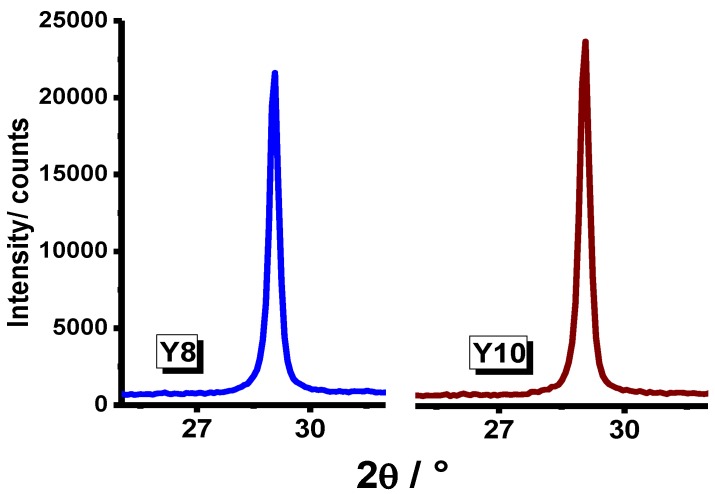
XRD pattern of Y_2_O_3_ powder synthesized from yttrium nitrate and yttrium chloride using P-123 poloxamer in a molar ratio of P-123:Y = 2:1.

The XRD results showed that Y_2_O_3_ systems embedded in P-123 poloxamer in a molar ratio of P-123:Y = 2:1 have presumably a better degree of crystallization, as shown in Figure 6. The crystallite sizes were determined by means of the Scherrer equation.

**Figure 7:**
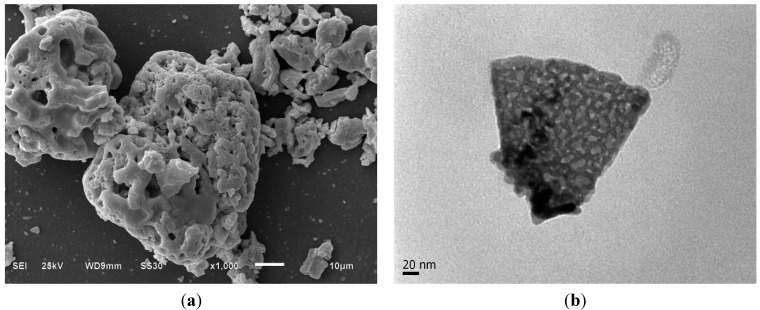
(**a**) Scanning electron microscopy (SEM) and (**b**) transmission electron microscopy (TEM) images of Sample Y-12 at 1000× and 300,000×, respectively.

Table 1 shows the sizes, in a range from 21 nm to 32 nm. The Sample Y-10 was observed by means of scanning electron microscopy (SEM) and transmission electron microscopy (TEM). SEM images of Sample Y-10 is presented in Figure 7a; a porous structure can be observed consequence of the incorporation of P-123 into the sol. The TEM image shown in Figure 7b reveals an aggregate of nanoparticles with an average size of about 25 nm in agreement with estimated by XRD.

### 2.2. Antioxidant Assays

**Figure 8:**
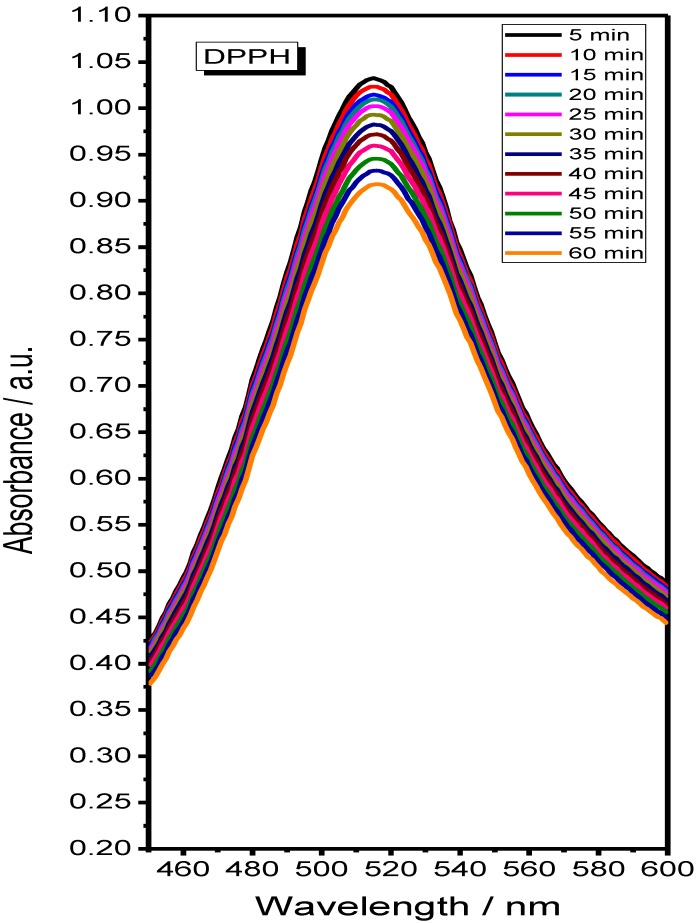
Time dependency of 2,2-diphenyl-1-picrylhydrazyl (DPPH•) scavenging in the absence of nanocrystals.

The DPPH• without nanoparticles does not reveal changes in the characteristic absorption peak (Figure 8). Antioxidant properties for Y_2_O_3_ Samples Y1–Y10 were tested. It is known that the antioxidant property may be due to the neutralization of DPPH• free radical character, explained by electron transference from the reactant [23].

**Figure 9:**
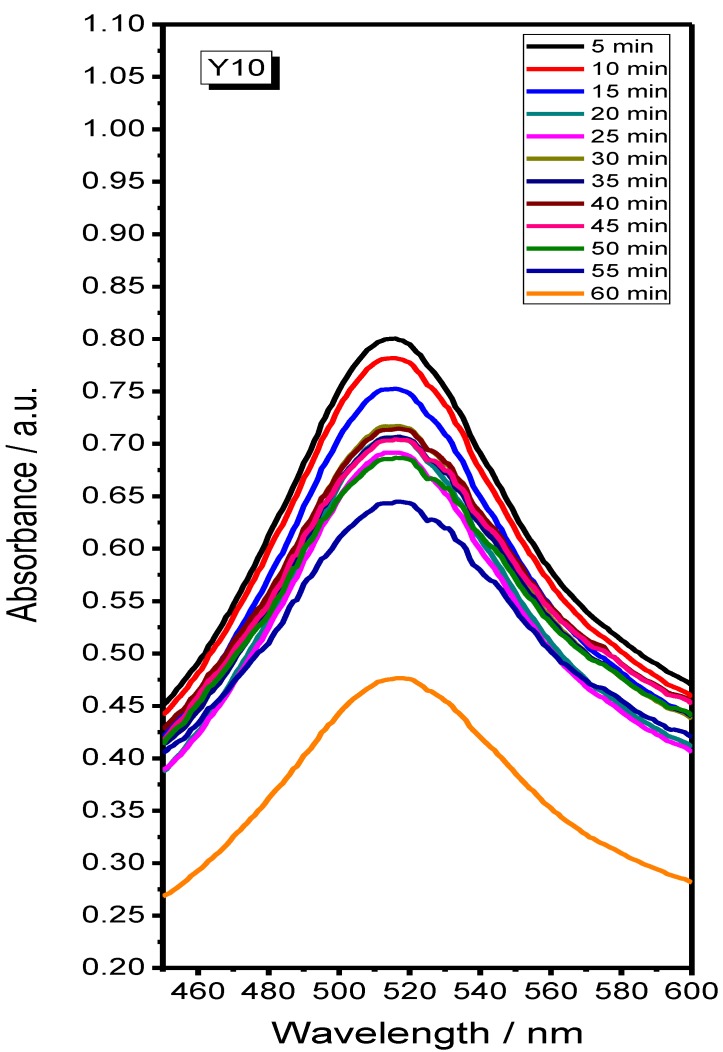
Time dependency of DPPH• scavenging in the presence of Y_2_O_3_ nanocrystallites (Sample Y10).

The graphic for Sample Y10 that showed the best antioxidant behavior of all synthesized compounds is presented in Figure 9. It can be observed that DPPH• diminishing begins at 5 min and it is evident after 60 min.

The percentage inhibition of the DPPH• radical was calculated according to the formula: %Inhibition = [*A*_C(0)_ – *A*_Y10(*t*)_/*A*_C(0)_] ° 100, where *A*_C(0)_ is the absorbance of the control, and *A*_Y10(*t*)_ is the absorbance in the presence of Y10 at different times (5–60 min).

The percentage inhibition of DPPH radical was 22.4% at 5 min and values varied in the range of times over which the reaction took place, showing 48.1% inhibition in a period of 60 min. The results demonstrate that Y10 reduces DPPH• to an extent and antioxidant activity increases with time reaching it highest value after one hour. As has been already observed in other materials, such iron oxide nanoparticles [24], the increment of the antioxidant activity is highly dependent of the particle size. This effect can be attributed to an increment on the surface area obtained from the proposed synthesis employing P-123.

## 3. Experimental Section

### 3.1. Synthesis Procedure

Y_2_O_3_ was prepared by sol-gel method according to the following procedure. All experiments were carried out at room temperature. Experimental strategies for Y_2_O_3_ powder preparation used two raw materials: yttrium nitrate and yttrium chloride. For Y_2_O_3_ synthesis hexa-hydrate yttrium nitrate (Y(NO_3_)_3_·6H_2_O, 99.8%, Sigma-Aldrich, St. Louis, MO, USA), hexa-hydrate yttrium chloride (YCl_3_·*x*H_2_O, 99.99%, Alfa Aesar, St. Louis, MO, USA), methanol (CH_3_OH (MeOH), 99.8%, Sigma-Aldrich), acetylacetone (CH_3_COCH_2_COCH_3_ (AcAc), 99.9%, Sigma-Aldrich), and for embedded system P-123 poloxamer ((C_3_H_6_O·C_2_H_4_O)_x_, molecular weight (MW): ~5800, Sigma-Aldrich) were used. 0.0026 mol of Y(NO_3_)_3_·6H_2_O or 0.0051 mol of YCl_3_·*x*H_2_O were dissolved in MeOH in a molar ratio of MeOH/Y = 123 under vigorous magnetic stirring for 15 min. Thereafter, AcAc was added in order to obtain a stable sol of Y_2_O_3_. In selected systems, P-123 poloxamer was incorporated in a molar ratio described in Table 1. Y_2_O_3_ xerogel was formed at 90 °C for 24 h. Finally, different heat-treatments were carried out in order to produce dense materials and to promote the crystallization of the Y_2_O_3_ powder at 270 °C (2 h), 500 °C, 700 °C, 800 °C and 900 °C for 1 h.

### 3.2. Apparatus

The FTIR transmittance spectra were recorded from Y_2_O_3_ powders heat-treated at different temperatures using a Bruker Tensor 27 Model (Billerica, MA, USA) in the range of 4000–400 cm^−1^. Samples were analyzed using the KBr pelleting technique (4.0 cm^−1^ resolution, 15 scans, 15 s). The structure was determined by a Bruker Diffractometer D8 Advance using a copper anticathode at 35 kV and 25 mA in a range 2θ from 15° to 80°. The crystallite size was determined according to the Scherrer equation, considering the line broadening of the diffraction peak due to the effect of crystal size [25]:
(1)
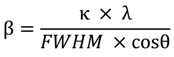

where β is the crystallite size of the powder, κ is the factor shape of the crystal, λ (0.15406 nm) is radiation wavelength, *FWHM* is the full-width at half-maximum of the peak, and θ is the Bragg angle of XRD peak. The microstructure of powders was determined by a SEM and TEM by means of JEOL JSM-6510LV and JEM 1400 Equipment (Tokyo, Japan), respectively.

## 4. Conclusions

Y_2_O_3_ nanocrystallites were successfully synthesized by sol-gel method from yttrium nitrate and yttrium chloride as precursors. Y_2_O_3_ nanostructured powders prepared from yttrium chloride and in the presence of P-123 poloxamer in a molar ratio of P-123:Y = 2:1 presented better physicochemical properties (crystallinity and purity) than systems prepared from yttrium nitrate precursor. Y_2_O_3_ powders presented a crystallite size range of 21 nm to 32 nm in agglomerate arrays. The DPPH• studies are reported for the first time for Y_2_O_3_ synthesized by sol-gel method, thus, there is no reference to compare. Y_2_O_3_ nanocrystallites in presence of P-123 compose of porous morphologies integrated with nanosized particles showed enhanced antioxidant properties, therefore they can be considered as new promising materials in biological systems.
